# Higher CSF Tau Levels Are Related to Hippocampal Hyperactivity and Object Mnemonic Discrimination in Older Adults

**DOI:** 10.1523/JNEUROSCI.1279-19.2019

**Published:** 2019-10-30

**Authors:** David Berron, Arturo Cardenas-Blanco, Daniel Bittner, Coraline D. Metzger, Annika Spottke, Michael T. Heneka, Klaus Fliessbach, Anja Schneider, Stefan J. Teipel, Michael Wagner, Oliver Speck, Frank Jessen, Emrah Düzel

**Affiliations:** ^1^Institute of Cognitive Neurology and Dementia Research (IKND), Otto-von-Guericke University, 39120 Magdeburg, Germany,; ^2^German Center for Neurodegenerative Diseases (DZNE), 39120 Magdeburg, Germany,; ^3^Clinical Memory Research Unit, Department of Clinical Sciences Malmö, Lund University, 223 62 Lund, Sweden,; ^4^Department of Neurology, University Hospital of Bonn, 53127 Bonn, Germany,; ^5^German Center for Neurodegenerative Diseases (DZNE), 53127 Bonn, Germany,; ^6^Department of Neurodegeneration and Geriatric Psychiatry, University of Bonn, 53127 Bonn, Germany,; ^7^Department of Neurology, University Hospital Magdeburg, 39120 Magdeburg, Germany,; ^8^Department of Psychiatry and Psychotherapy, Otto-von-Guericke University, 39120 Magdeburg, Germany,; ^9^Department of Psychosomatic Medicine, Rostock University Medical Center, 18147 Rostock, Germany,; ^10^German Center for Neurodegenerative Diseases, 18147 Rostock, Germany,; ^11^Department of Biomedical Magnetic Resonance, Otto-von-Guericke University, 39120 Magdeburg, Germany,; ^12^Department of Psychiatry, University Hospital Cologne, 50937 Cologne, Germany,; ^13^University College London, Institute of Cognitive Neuroscience, London WC1N 3AZ, United Kingdom,; ^14^Leibniz Institute of Neurobiology, 39120 Magdeburg, Germany, and; ^15^Center for Behavioral Brain Sciences, 39120 Magdeburg, Germany

**Keywords:** ageing, CSF, entorhinal cortex, fMRI, hippocampus, mnemonic discrimination

## Abstract

Mnemonic discrimination, the ability to distinguish similar events in memory, relies on subregions in the human medial temporal lobes (MTLs). Tau pathology is frequently found within the MTL of older adults and therefore likely to affect mnemonic discrimination, even in healthy older individuals. The MTL subregions that are known to be affected early by tau pathology, the perirhinal-transentorhinal region (area 35) and the anterior-lateral entorhinal cortex (alEC), have recently been implicated in the mnemonic discrimination of objects rather than scenes. Here we used an object-scene mnemonic discrimination task in combination with fMRI recordings and analyzed the relationship between subregional MTL activity, memory performance, and levels of total and phosphorylated tau as well as Aβ42/40 ratio in CSF. We show that activity in alEC was associated with mnemonic discrimination of similar objects but not scenes in male and female cognitively unimpaired older adults. Importantly, CSF tau levels were associated with increased fMRI activity in the hippocampus, and both increased hippocampal activity as well as tau levels were associated with mnemonic discrimination of objects, but again not scenes. This suggests that dysfunction of the alEC-hippocampus object mnemonic discrimination network might be a marker for tau-related cognitive decline.

**SIGNIFICANCE STATEMENT** Subregions in the human medial temporal lobe are critically involved in episodic memory and, at the same time, affected by tau pathology. Impaired object mnemonic discrimination performance as well as aberrant activity within the entorhinal-hippocampal circuitry have been reported in earlier studies involving older individuals, but it has thus far remained elusive whether and how tau pathology is implicated in this specific impairment. Using task-related fMRI in combination with measures of tau pathology in CSF, we show that measures of tau pathology are associated with increased hippocampal activity and reduced mnemonic discrimination of similar objects but not scenes. This suggests that object mnemonic discrimination tasks could be promising markers for tau-related cognitive decline.

## Introduction

Memory for objects and scenes relies on different processing pathways in the human brain (Ranganath and Ritchey, 2012; Ritchey et al., 2015). Whereas memory for scenes is based on the posterior-medial system, memory for objects mainly depends on regions in the anterior-temporal system. Studies in rodents suggest that the two cortical systems extend toward the medial temporal lobe (MTL) where the lateral entorhinal cortex is more involved in object memory and processing of local landmarks, whereas the medial entorhinal cortex (MEC) is critical for spatial memory and processing of global landmarks (Knierim et al., 2014). Recent research could translate these findings to humans and showed that the perirhinal cortex and the anterior-lateral EC (alEC) are specifically involved in object memory, whereas the parahippocampal cortex and the posterior-medial EC (pmEC) are preferentially involved in spatial and scene memory (Reagh and Yassa, 2014; Maass et al., 2015; Schröder et al., 2015; Berron et al., 2018).

It has been suggested that aging predominantly affects the object memory pathway, and there are indeed several recent studies showing evidence for early impairment in object memory with age (Ryan et al., 2012; Fidalgo et al., 2016; Reagh et al., 2016; Olsen et al., 2017; Berron et al., 2018). Difficulties in object memory have been shown to be associated with reduced volume or lesions of the alEC (Olsen et al., 2017; James et al., 2018) and reduced BOLD activity in alEC and perirhinal cortex (Ryan et al., 2012; Reagh et al., 2018). Using mnemonic discrimination (Bakker et al., 2008; Lacy et al., 2011; Berron et al., 2016) in a novel task with isolated objects and scenes, we found that objects and scenes did not elicit equivalent levels of activity in older adults and that this imbalance was related to performance in discriminating very similar object pairs from memory (Berron et al., 2018). Mnemonic discrimination tasks have in common that they likely pose high demands on pattern separation functions, which have been suggested to rely on the inputs from the lateral and medial EC to the dentate gyrus (DG). Together, these findings indicate that discriminating objects with high feature overlap in memory may be particularly vulnerable to aging.

Age-related decline of mnemonic discrimination in older adults that do not show mild cognitive impairment (MCI) could be due to aging or due to incipient, preclinical Alzheimer's disease (AD) pathology (Jack et al., 2018). AD is characterized by β-amyloid (Aβ) and tau pathology (Hyman et al., 2012). Aβ accumulates early and predominantly in the retrosplenial, posterior cingulate and mPFC (Palmqvist et al., 2017). Tau pathology, on the other hand, can predominantly be found in the transentorhinal region (which corresponds to area 35 [A35] of the perirhinal cortex) and the alEC in early disease stages (Braak and Braak, 1991; Bennett et al., 2006). Thus, the pattern of tau pathology in preclinical AD stages overlaps to a substantial degree with the object pathway that has been shown to be especially involved in object mnemonic discrimination tasks. However, although there is convincing regional overlap of domain-specific memory functioning and early deposition of AD pathology, it remains unclear whether and how Aβ and tau pathology relates to the observed difficulties in mnemonic discrimination of objects and scenes as well as alterations in neural functioning.

Here we addressed this by analyzing the relationship of tau and amyloid pathology measured in CSF taps and the performance in a novel mnemonic discrimination task, which has been shown to tax object and scene processing pathways in the MTL, respectively (Berron et al., 2018). To explore the underlying neuronal dysfunction, we analyzed task-related fMRI activity in MTL subregions that are vulnerable to early AD, such as the transentorhinal region (corresponding to A35) and alEC, during the mnemonic discrimination task and tested its differential relationship on Aβ and tau pathology, respectively.

## Materials and Methods

### 

#### 

##### Overall study design.

DELCODE is an observational longitudinal memory clinic-based multicenter study in Germany. The detailed study design of DELCODE was reported by Jessen et al. (2018). The participants to be enrolled are 400 subjects with subjective cognitive decline (SCD), 200 patients with MCI, 100 AD dementia patients, 200 healthy controls without subjective or objective cognitive decline, and 100 first-degree relatives of patients with a documented diagnosis of AD dementia. All patient groups (SCD, MCI, AD) are referrals, including self-referrals, to the participating memory centers. The controls and the first-degree relatives of AD dementia patients are recruited by standardized public advertisement. SCD is defined by the presence of subjectively reported decline in cognitive functioning with concerns and a test performance within 1.5 SDs below the age-, sex-, and education-adjusted normal performance on all subtests of the CERAD neuropsychological battery. The control group and the group of first-degree relatives of AD patients were recruited by identical local newspaper advertisements. In the advertisement text, individuals were explicitly sought who felt healthy and without relevant cognitive problems. All individuals who responded to the advertisement were screened by telephone with regard to SCD. The report of very subtle cognitive decline, which did not cause any concerns and was considered normal for age by the individual, was not an exclusion criterion for the control group. For the first-degree relatives of AD dementia, the advertisement did not exclude those with concerns of cognitive decline. AD dementia in the relative (parent or sibling) had to be documented by medical records. Both the control group and the group of first-degree relatives had to achieve unimpaired cognitive performance according to the same definition as the SCD group.

Additional inclusion criteria for all groups were age ≥ 60 years, fluent German language skills, capacity to provide informed consent, and presence of a study partner. For definitions of AD dementia and MCI, not pertinent to the present paper, and general exclusion criteria, see Jessen et al. (2018). Ten university-based memory centers are participating, which are all collaborators of local sites of the German Center for Neurodegenerative Diseases.

##### Clinical, neuropsychological, and risk factor assessments.

Within DELCODE, the clinical and neuropsychological assessments were performed at baseline by a trained study physician (clinical scales) or clinical neuropsychologist (cognitive testing) (for a detailed overview, see Jessen et al., 2018). The order of examinations was fixed. All examinations were performed within 1 d. The clinical assessments included a structured medical history, current medication, structured family history, standardized physical examination including sensory testing, the Mini Mental State Examination, the Clinical Dementia Rating, the 16-item short form of the Geriatric Depression Scale, the short form of the Geriatric Anxiety Inventory, the Neuropsychiatric Inventory, and the Functional Activities Questionnaire. Depression and substance use were assessed in a standardized fashion according to ICD-10. The cognitive test battery included, among others, the ADAScog 13 and the Free and Cued Selective Reminding Test. The results of these assessments for the first 394 individuals are reported by Jessen et al. (2018).

##### Participants.

For this paper, we analyzed data from cognitively unimpaired individuals (healthy controls, first-degree relatives, and patients with SCD), who had participated in a task fMRI session on object and scene mnemonic discrimination and who consented to a CSF tap. The object and scene fMRI task was performed in Bonn, Magdeburg, and Rostock. Two participants had to be excluded in the beginning due to chance performance. As a result, 21 cognitively unimpaired participants were analyzed (Table 1). Those consisted of 14 healthy controls (mean age = 65 years; SD = 3.5 years; 7 female), 2 first-degree relatives to AD patients (mean age = 62 years; SD = 2.8 years; 1 female), and 5 individuals with SCD (mean age = 70 years; SD = 2.9 years; 4 female). Normal and corrected vision was assessed using standard procedures and printed stimulus materials comparable to the materials used during the experiments. The study was conducted and designed in accordance with the Declaration of Helsinki (Williams, 2008), and all subjects gave informed and written consent for their participation. The study protocol was approved by the ethical committees of the medical faculties of Bonn, Magdeburg, and Rostock. The process was led and coordinated by the ethical committee of the medical faculty of the University of Bonn. The registration number of the trial at the ethical committee in Bonn is 117/13.

**Table 1.** Participant information*^a^*

Characteristic	Value
*N*	21
Gender	12 female/9 male
Age (yr)	66.1 (3.9)
CSF p-tau	52.1 (15.1)
CSF t-tau	374.5 (115.9)
CSF Aβ42/40	0.095 (0.022)
MMSE	29.4 (1)
FCSRT-free	31.7 (6.5)
FCSRT-cued	16.2 (6.4)
ADAS-cog delayed recall	8.2 (1.7)

*^a^*Data are mean (SD). MMSE, Mini Mental State Examination; FCSRT, Free and Cued Selective Reminding Test; ADAS-cog, Alzheimer's Disease Assessment Scale-cognitive subscale.

##### CSF AD biomarker assessment.

CSF samples were collected from all 21 participants. More details of the assessment are reported by Jessen et al. (2018). AD biomarkers were determined centrally at the Bonn site using commercially available kits according to vendor specifications (V-PLEX Aβ Peptide Panel 1, 6E10 Kit, K15200E and V-PLEX Human Total Tau Kit, K151LAE, Mesoscale Diagnostics; and Innotest Phospho Tau(181P), 81581, Fujirebio Germany). CSF samples were tested in duplicates with a coefficient of variance < 20%. An internal control sample was used with each assay plate to ensure the general performance of the assay and that results are within the correct range. The laboratory furthermore participates in the Alzheimer's Association Quality Control program for AD biomarkers ensuring continuity of test results.

##### MRI acquisition.

MRI data were acquired at 3 scanning sites operating Siemens 3 tesla (T) MRI scanners, including a 3T MAGNETOM Trio system, a 3T MAGNETOM Verio system, and a 3T MAGNETOM Skyra system, all of them equipped with 32-channel phased array imaging coils used in receive mode. The MR protocol included a T1-weighted high-resolution sequence obtained using a 3D MPRAGE sequence with the following parameters: TE/TR = 4.3/2500 ms, inversion time = 1100 ms, flip angle = 7 degrees, receiver bandwidth = 140 Hz/Px, distance factor = 50%, 192 slices of 1 mm thickness, and a matrix size of 256 × 256, yielding an isotropic resolution of 1 mm^3^. We allowed for parallel acquisition of independently reconstructed images using generalized, autocalibrating, partially parallel acquisitions or GRAPPA (Griswold et al., 2002) with acceleration factor 2 and 24 reference lines with a total acquisition time of 5 min 8 s. Identical MR protocols were used at all sites.

An inversion recovery EPI used the following parameters: TE/TR = 40/6100 ms, inversion time = 1100 ms, receiver bandwidth = 1718 Hz/Px, distance factor = 10%, 36 slices of 3.4 mm thickness, and a matrix size of 104 × 104, yielding a resolution of 2 × 2 × 3.4 mm. The acquisition was accelerated using GRAPPA, factor 2 and 24 reference lines with a total acquisition time of 0 min 26 s.

A two-echoes, gradient echo sequence was acquired to estimate the B0-fieldmap using the following parameters: TE/TR = 4.92 and 7.38 ms/675 ms, flip angle = 60 degrees, receiver bandwidth = 327 Hz/Px, distance factor = 10%, 36 slices of 3.4 mm thickness, and a matrix size of 104 × 104, yielding a resolution of 2 × 2 × 3.4 mm with a total acquisition time of 2 min 23 s.

A 3D T2-weighted structural turbo spin echo scan optimized for volumetric assessment of the MTL acquired in oblique coronal orientation perpendicular to the longitudinal axis of the hippocampus using the following parameters: TE/TR = 353/3500 ms, receiver bandwidth = 434 Hz/Px, 64 slices of 1.5 mm thickness, and a matrix size of 384 × 384, yielding an in-plane resolution of 0.5 × 0.5 mm. The acquisition was accelerated using GRAPPA, factor 2 and 24 reference lines with a total acquisition time of 11 min 45 s.

An EPI sequence was used to measure the BOLD response using the following parameters: TE/TR = 30/2400 ms, flip angle = 80 degrees, 300 volumes, receiver bandwidth = 1718 Hz/Px, distance factor = 10%, 36 slices of 3.4 mm thickness, and a matrix size of 104 × 104, yielding a resolution of 2 × 2 × 3.4 mm. The acquisition was accelerated using GRAPPA, factor 2 and 24 reference lines with a total acquisition time of 12 min 9 s.

##### Stimuli and setting.

**Figure 1.**
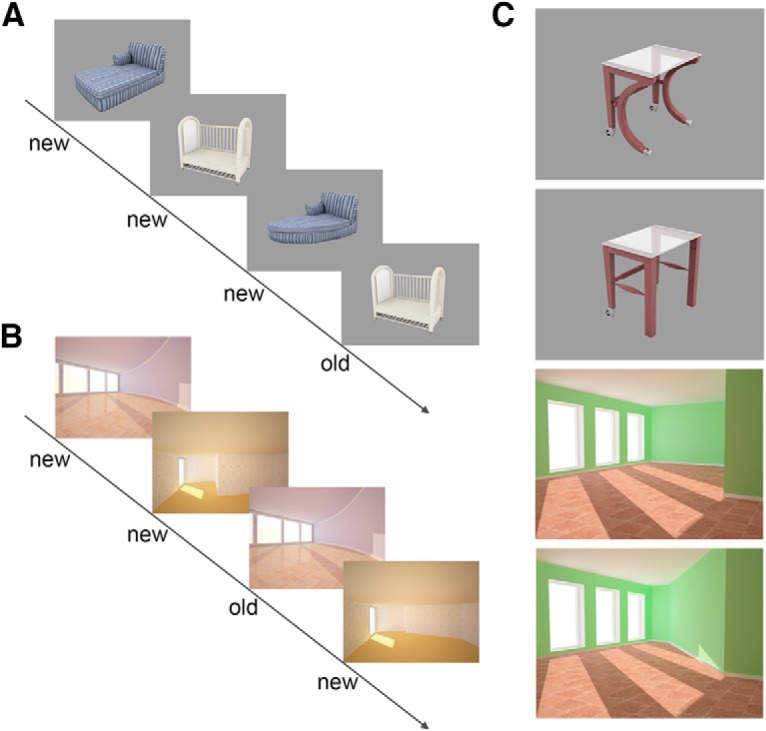
Stimulus sequence and example stimuli. Participants see sequences with object (***A***) and scene pairs (***B***). For every image, they have to decide whether the image is old (repeated) or new. They press “new” whenever an image is entirely new (first presentation) or a similar version of an earlier presentation (lure). The similar object and scene pairs have been generated by changing local features of the object or global boundaries of the rooms (***C***). Reprinted with permission from Berron et al. (2018, their Fig. 1).

Stimuli consisted of computer-generated (3 ds Max, Autodesk) and isoluminant images. The images were comprised of everyday indoor objects presented on a gray background as well as empty indoor scenes (empty rooms; Fig. 1*C*). Lure stimuli were created by changing the local features of the objects (shape of the table leg; Fig. 1*C*) but not color, position, or size of the objects, and the global boundary features of the rooms (geometry of the empty room, but again not the color or viewpoint). Each room and object was presented two times where the second presentation was either an identical (repeats) or the lure version (lures) (Fig. 1*A*,*B*). Task difficulty of discriminating lures and repeats was matched across object and scene conditions with respect to the mean and variance. The fixation target was a white fixation star.

For task fMRI, all sites were equipped with a high-resolution (but set to 1280 × 800 Px) 30 inch MR-compatible LCD screen (Medres Optostim). All monitors were calibrated and configured to maintain the distance, luminance, color, and contrast constant across sites. Responses during task fMRI were recorded at all sites with MR-compatible response buttons (CurrentDesign). All participants underwent vision correction with MR-compatible goggles (MediGlasses, Cambridge Research Systems) according to the same standard operating procedure for all MRI sites. For experimental presentation, we used Presentation Software (Neurobehavioral Systems; https://nbs.neurobs.com). Subjects' positioning in the MRI scanner as well as sequence preparation steps (e.g., image angulation) were standardized across all sites by provided standard operating procedures and on-site training.

##### Task and experimental design.

We used the same task that we have explained earlier in detail (Berron et al., 2018). Before scanning, subjects were instructed verbally and saw a standardized visual instruction with all information regarding the experiment. Subsequently, they had to learn the task within a 5 min training session outside the scanner. In addition, a standard vision screening procedure as well as a visual discrimination test with stimuli comparable to the ones used in the experiment were conducted to rule out possible confounding effects of a deficit in visual perception (Davidson et al., 2019). In a control analysis, Pearson correlation coefficients were calculated to assess the possible relationship between lure discrimination and the participants' individual vision. However, this analysis did not yield any significant relationship for object (*r* = 0.019, *p* = 0.936) or scene mnemonic discrimination (*r* = −0.046, *p* = 0.843). During the following fMRI session, stimuli were presented in sequences of four stimuli (Fig. 1*A*,*B*) of either objects or scenes (for details, see Berron et al., 2018). The first two stimuli of a sequence were always new images, whereas the following two could be either an exact repetition (repeat) or a very similar version of the previous ones (lure). Stimuli were presented in an event-related design, in which each stimulus was shown for 3 s and stimuli were separated by a fixation star. Interstimulus intervals, ranging from 0.6 to 4.2 s (mean 1.63 s), were jittered to optimize statistical efficiency (Dale, 1999). Intervals between sequences were longer (mean 2.43 s) to indicate the end of a sequence. Subjects had to respond to each stimulus with old/new judgments using their right index and middle finger. Subjects were told to press “new” for entirely new images but also for very similar versions of earlier images. “Old” responses should be given for exact repetitions. The presentation of sequences was counterbalanced with respect to objects and scenes as well as repeats and lures. Thus, a sequence could consist of only objects or scenes, but it was unpredictable for a subject whether there will be a lure or repeat pair as all possible combinations (i.e., lure-lure, repeat-repeat, repeat-lure, and lure-repeat) were counterbalanced. This resulted in a total of 56 sequences (where each sequence consists of 4 stimuli) across both domains with 56 first-repeat pairs and 56 first-lure pairs, which add up to a total of 224 object and scene trials. At the beginning and the end of each sequence, we presented 10 scrambled images of earlier presented objects and scenes to have a baseline with comparable low-level sensory information but without the meaning and semantics associated with the actual objects or scenes, which, however, has not been used in the analyses.

##### Behavioral data analysis.

Accuracy scores were analyzed using SPSS 24 (IBM). Hit rates (repeats percent correct), correct rejection rates (lures percent correct), false alarm rates (lures percent incorrect), and corrected hit rates (hit rates minus false alarm rates) were calculated for the object and scene condition and *z*-standardized across different sites. We performed an ANOVA to test for differences in task accuracy with two within-subject factors task-condition (hit rate, correct rejection rate) and domain (object, scene). Throughout the manuscript, we use the term “mnemonic discrimination performance” to refer to the correct rejection rate.

##### fMRI data analyses.

For preprocessing and statistical analyses, we used the Statistical Parametric Mapping software (SPM, version 12; Wellcome Trust Centre for Neuroimaging). All functional images were corrected for differences in the time of slice acquisition and were realigned to the first image of the first session following motion estimation. The anatomical T1 image was coregistered to the mean functional image. Functional images were spatially smoothed using an isotropic Gaussian kernel of FWHM 4 × 4 × 4 mm with the purpose of increasing the signal-to-noise ratio (SNR). Images were high-pass filtered (128 s) to remove low-frequency signal drifts. We used a first-order autoregressive model for estimating temporal autocorrelations by using restricted maximum likelihood estimates of variance components. To model the functional data, δ functions defined by the onset of a stimulus on a trial-by-trial basis were convolved with an HRF. First-level data were analyzed using a mixed-effects GLM approach. All experimental conditions were entered into the GLM as separate regressors for the following conditions: first presentations, repeats, correct lures and incorrect lures separately for objects and scenes as well as scrambled images (i.e., 9 conditions in total). Data from the first and second run were concatenated using the *spm_fmri_concatenate.m* function in SPM12. Using this function, the high-pass filtering and prewhitening were applied on a session-specific basis to account for the original session lengths. Furthermore, six motion parameters were added as regressors of no interest to minimize false-positive activations due to task correlated motion (Johnstone et al., 2006). At a single subject level, contrasts were created by comparing all scene and object trials (scene firsts, repeats and lures > object firsts, repeats and lures; and vice versa). To include all voxels in the MTL, an explicit mask involving gray and white matter as well as CSF was used in SPM12.

##### Automated segmentation of subregions in the MTL.

**Figure 2.**
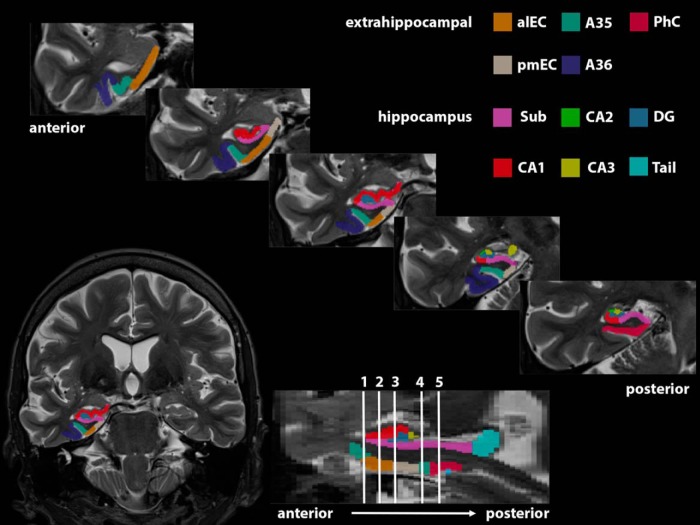
Result of the combined automated and manual segmentation of subregions in the MTL. Result of the segmentation of extrahippocampal subregions and hippocampal subfields are displayed in five slices from anterior to posterior. alEC, anterior-lateral entorhinal cortex; pmEC, posterior-medial entorhinal cortex; A35, area 35; A36, area 36; PhC, parahippocampal cortex; Sub, subiculum; CA, cornu ammonis; DG, dentate gyrus.

The MTLs were subdivided in subregions using a three-level approach. First, we used the package for automated segmentation of hippocampal subfields (Yushkevich et al., 2015) to automatically segment the MTL in the perirhinal cortex, consisting of separate labels for A35 and area 36, parahippocampal cortex, entorhinal cortex, and hippocampal subfields, namely, subiculum, cornu ammonis (CA) 1–3, and DG. We used an atlas set, which is available within the automated segmentation of hippocampal subfields package and was trained on manual segmentations of 38 individuals (Magdeburg 7T atlas) (for further explanation, see Berron et al., 2017). In a second step, all segmentations were manually checked and corrected where this was necessary, and hippocampal subfields were merged to one hippocampal mask as our functional resolution did not allow for detailed analyses of activation within hippocampal subfields. In a third step, to get masks of subregions in the EC, namely, alEC and pmEC, we used the masks that are available online from Maass et al. (2015) as a visual guide. To that end, we coregistered the alEC/pmEC masks to our study-specific template and followed a template-based approach using ANTs to transform alEC and pmEC masks from the study-specific template to the native space T2 image of each participant (Avants et al., 2010). alEC/pmEC masks were overlaid on the whole EC masks derived from the automated segmentation as a visual guide with the aim to manually subdivide whole EC masks in alEC and pmEC portions (Fig. 2). Finally, native T2 images were coregistered to the T1 images using ANTs to be able to transfer masks of MTL subregions between native T2 and native EPI space. The subregional masks were manually checked for their correct location following the transformation.

##### ROI analysis.

For ROI analyses, we extracted mean *t* values from anatomically defined masks in the MTL and estimates from different MRI sites were *z*-normalized. To investigate domain specificity within MTL regions, we extracted domain-specificity scores from the scenes > objects contrast (scenes − objects), which we refer to as domain-specificity score (Berron et al., 2018). Positive domain-specificity scores indicate higher activation for scenes, whereas negative scores reflect higher activation for objects. We also investigated lure-related activation by contrasting lure stimuli against first presentations (Bakker et al., 2012; Reagh et al., 2018). Individual anatomical masks were thresholded using the implicit mask in SPM12 (0.8) before the ROI analysis. This was done to delete voxels affected by signal drop-out from the anatomical masks.

As we used data from different sites with different MR scanners, one potential concern could be data quality differences across scanners which might drive the results. Thus, we calculated temporal SNR (tSNR) as the mean intensity in a given voxel of the time series divided by its SD for the entorhinal cortex. The entorhinal cortex had a mean tSNR of 11 across all sites and was fairly similar between sites (site 1: mean = 9, SD = 0.7, *n* = 6; site 2: mean = 12, SD = 5.6, *n* = 12; site 3: mean = 10, SD = 0.2, *n* = 3). Furthermore, to rule out that subjects with lower tSNR could be driving the effects, we analyzed whether there was a relationship of tSNR in alEC and the resulting activity estimates. There was no relationship between tSNR and activity estimates either in the left or in the right alEC (left: *r* = 0.164, *p* = 0.503; right: *r* = −0.039, *p* = 0.874).

##### Statistical analyses.

Partial correlation analyses in SPSS 24 were used to assess relationships between mnemonic discrimination performance, BOLD activation, and CSF measures of tau (p-tau and t-tau) and Aβ (Aβ42/40) pathology. ggplot2 in R version 3.3.2 (www.r-project.org) was used for visualization of the statistical relationships (Wickham, 2016). All correlation analyses were adjusted for age and sex. In addition, all correlation analyses, including fluid biomarkers, were additionally controlled for ventricle volume by including ventricle volume derived from a T1-based Freesurfer segmentation (Freesurfer 6.0; http://surfer.nmr.mgh.harvard.edu) as a covariate to account for differences in the overall amount of CSF (based on personal communication with Niklas Mattson). We applied the Bonferroni–Holm correction to correct for multiple comparisons where appropriate.

## Results

### Behavioral performance

**Figure 3.**
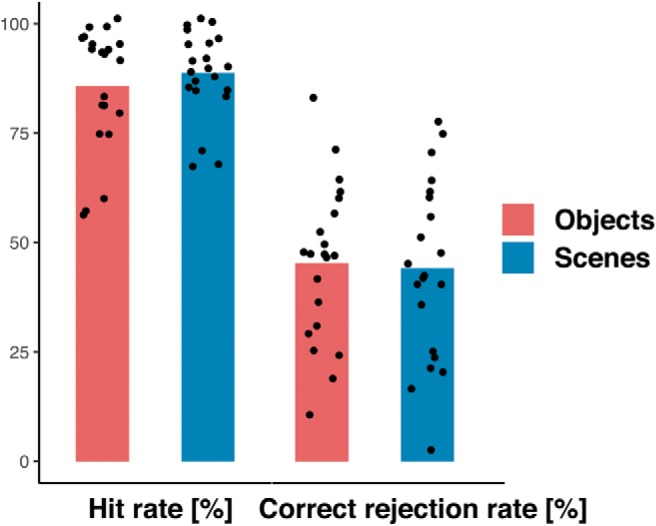
Behavioral performance in the object and scene mnemonic discrimination task.

For discrimination accuracies, an ANOVA with the within-subject factors domain (scene and object) and measure (hit rate and correct rejection rate) was performed. This ANOVA showed no significant main or interaction effects for the domain, suggesting that there was no difference in hit rates and correct rejection rates between the scene and object condition (scenes: M_HR_ = 85.7, M_CRR_ = 45.2; objects: M_HR_ = 88.8, M_CRR_ = 44) (Fig. 3).

### CSF measures of tau, but not amyloid, are associated with object mnemonic discrimination performance

**Figure 4.**
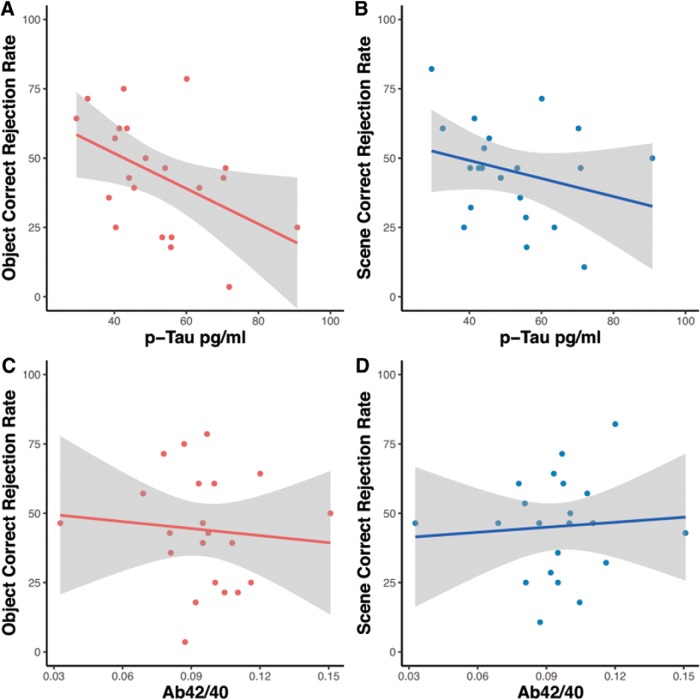
Relationship of object and scene mnemonic discrimination performance and CSF measures of Aβ and tau.

With respect to our main hypothesis that biomarker pathology would be associated with object mnemonic discrimination performance, we set out to test whether CSF measures of Aβ and tau were associated with object and scene correct rejection rates, respectively. Partial correlation analyses revealed that both CSF measures of phosphorylated tau (p-tau) and total tau (t-tau) were significantly associated with individual object (*r* = −0.641, *p* = 0.004; *r* = −0.480, *p* = 0.044; corrected for multiple comparisons) but not scene mnemonic discrimination scores (correct rejection rates) (*r* = −0.360, *p* = 0.142; *r* = −0.267, *p* = 0.284) (Fig. 4*A*,*B*). Furthermore, the correlation of p-tau and object discrimination performance was significantly stronger than with scene discrimination (*z* = 1.9, *p* = 0.029) (Lenhard and Lenhard, 2014). However, neither object nor scene discrimination performance showed a significant relationship with CSF Aβ42/40 (*r* = −0.036, *p* = 0.888; *r* = 0.135, *p* = 0.595) (Fig. 4*C*,*D*).

### Reduced domain-specificity in alEC and A35 is associated with mnemonic discrimination of similar objects

**Figure 5.**
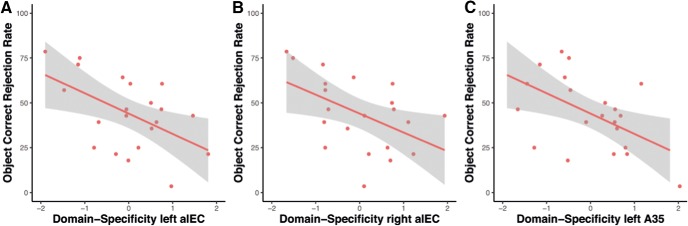
Association of domain-specific activity and object mnemonic discrimination performance.

Next, we wanted to find out whether functional activity in the MTL is associated with the observed tau-related object mnemonic discrimination performance. MTL subregion A35 (corresponding to the transentorhinal region) and alEC are among the earliest brain regions that show tau accumulation. Thus, we went on to test whether task-related activation in these regions would be associated with tau pathology and object and scene mnemonic discrimination performance. In a prior study, we found that domain-specific activation (meaning scene- minus object-related activation) in the perirhinal cortex, including A35, was related to impairment in object mnemonic discrimination in healthy older adults (Berron et al., 2018). Here, we were able to replicate these findings by showing that domain-specific activation in bilateral alEC was associated with reduced object discrimination performance (left: *r* = −0.494, *p* = 0.031; right: *r* = −0.496, *p* = 0.031; only the right side survives multiple comparison correction) and by observing a trend for left A35 (left: *r* = −0.426, *p* = 0.069; right: *r* = −0.3, *p* = 0.212) (Fig. 5). However, there was no significant relationship between alEC domain-specific activation and scene mnemonic discrimination (left: *r* = −0.335, *p* = 0.161; right: *r* = −0.093, *p* = 0.704). In a further step, we also tested whether the observed reduced domain specificity in alEC was associated with CSF measures of Aβ and tau. However, we did not find a significant relationship of measures of domain-specific activation in alEC with measures of p-tau (*r* = 0.088, *p* = 0.730; *r* = −0.034, *p* = 0.894) or Aβ42/40 (*r* = 0.175, *p* = 0.487; *r* = 0.249, *p* = 0.319).

To make sure that our results are not driven by the potential blurring of activity from A35, we performed a rather conservative additional analysis. We applied the same smoothing to A35 anatomical masks as we did to the functional data, thus mirroring the exact same blurring effects. In a second step, we deleted all voxels from the alEC masks that overlapped with the smoothed A35 masks and still found almost the same significant relationships between object discrimination performance and domain-specific signal in alEC (left: *r* = −0.487, *p* = 0.035; right: *r* = −0.502, *p* = 0.029).

### CSF p-tau is associated with increased hippocampal activation, which is in turn associated with mnemonic discrimination of objects

**Figure 6.**
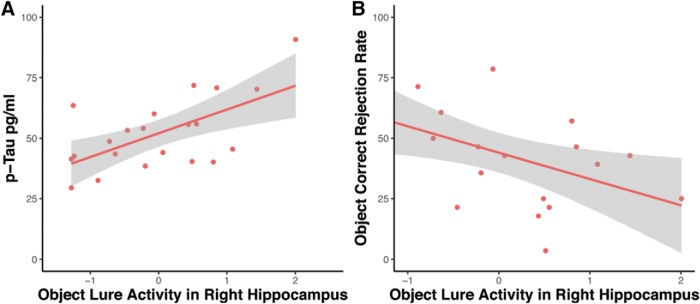
Relationship of increased hippocampal activation and object mnemonic discrimination performance as well as p-tau levels in CSF.

Earlier studies found that increased hippocampal activation (also referred to as hippocampal hyperactivity) during lure judgments was related to lower performance in object mnemonic discrimination (Bakker et al., 2012, 2015; Reagh et al., 2018). Thus, we tested whether elevated levels of CSF p-tau were associated with increased hippocampal activation. Our results show that there was a positive correlation between CSF p-tau levels and increased right hippocampal activation (*r* = 0.545, *p* = 0.019; corrected for multiple comparisons; Fig. 6*A*). This was not true for the left hippocampus (*r* = −0.034, *p* = 0.895), and bilateral hippocampal activation during scene lure judgments was also not significantly related to CSF p-tau (left: *r* = −0.163, *p* = 0.518; right: *r* = −0.309, *p* = 0.212). Given the relationship of p-tau and the increased right hippocampal activation, we further tested whether object mnemonic discrimination performance was significantly associated with hippocampal activation. We found that right hippocampal activation during correct object lure judgments was indeed negatively associated with object mnemonic discrimination performance (*r* = −0.486, *p* = 0.035; Fig. 6*B*). Again, this was not true for scene mnemonic discrimination performance (left: *r* = −0.289, *p* = 0.231; right: *r* = −0.315, *p* = 0.190).

## Discussion

This study explored the relationship between Aβ and tau pathology measured in CSF, object and scene mnemonic discrimination performance, and task-related brain activity in cognitively unimpaired older adults. We found that CSF tau levels were significantly related to object but not scene mnemonic discrimination, although there was no relationship with Aβ levels. Although we found that reduced domain-specific activity in alEC was associated with object mnemonic discrimination performance, we did not find a relationship with CSF measures of tau or Aβ. However, CSF p-tau levels were associated with increased hippocampal activity, which in turn was associated with mnemonic discrimination of similar objects but not scenes.

### Effect on mnemonic discrimination of similar objects

Age-related impairment in mnemonic discrimination tasks has been reported by several earlier studies (Stark et al., 2013, 2015), where most tasks focused on mnemonic discrimination of similar objects (Toner et al., 2009; Yassa et al., 2011a,b). Several recent studies set out to investigate age-related effects using paradigms that allow to compare object and scene (or spatial) mnemonic discrimination (Fidalgo et al., 2016; Reagh et al., 2016; Stark and Stark, 2017; Berron et al., 2018). While all these studies report impairment in both domains, several suggest that object mnemonic discrimination might be more sensitive to aging than scene discrimination (Fidalgo et al., 2016; Reagh et al., 2016; Berron et al., 2018). The regional overlap of early tau pathology in MTL subregions with brain regions that are significantly involved in object processing (e.g., the entorhinal/perirhinal cortices) suggests that object memory tasks, especially those using similar stimulus pairs, might be sensitive measures for tau-related cognitive decline (Braak and Braak, 1991; Schöll et al., 2016; Das et al., 2018). Here we found a significant relationship between mnemonic discrimination performance of similar objects, but not scenes and individual levels of CSF p-tau and t-tau. In addition, a comparison of both correlations showed that the relationship of object mnemonic discrimination performance and CSF levels of p-tau is significantly stronger compared with scenes. This suggests that object discrimination performance is more tightly linked to tau burden in cognitively unimpaired older individuals despite being correlated with scene mnemonic discrimination performance. On the other hand, however, CSF Aβ42/40 levels in our cognitively unimpaired older participants were neither related to object nor to scene mnemonic discrimination performance.

### BOLD activity in MTL subregions is associated with mnemonic discrimination performance

Our finding that measures of tau pathology are associated with memory performance is in line with several recent studies (Marks et al., 2017; Düzel et al., 2018; Maass et al., 2018) and the general notion that tau pathology is more strongly associated with cognitive decline than Aβ (Nelson et al., 2012). However, CSF-based measures of tau pathology lack spatial specificity; thus, it is difficult to know whether tau pathology in the MTL is underlying this memory impairment. To study regional dysfunction in specific MTL subregions, we analyzed task-based fMRI recordings during the mnemonic discrimination task. Earlier studies highlighted activity in the EC (especially its anterior-lateral portion, alEC), the PRC and the hippocampus of older individuals to be related to mnemonic discrimination performance. Reagh et al. (2018) used a mnemonic discrimination task where participants had to discriminate similar objects and object locations. When comparing young and older individuals, they found that older individuals were characterized by diminished alEC activity and an impairment in object mnemonic discrimination with only subtle impairment in discriminating the object location (Reagh et al., 2018). Using our object-scene mnemonic discrimination task, we have recently shown that domain-specific activity (scene- vs object-related activity) in PRC and alEC was diminished in older compared with young participants and that an imbalance in domain-specific activity was related to impaired performance in the object mnemonic discrimination task (Berron et al., 2018). Here, we replicated this finding in a more detailed analysis by showing that domain-specific activity in alEC (and a trend in A35) was associated with object mnemonic discrimination in cognitively unimpaired older individuals. However, although we observed that both CSF p-tau levels and domain-specific MTL activity were associated with object mnemonic discrimination performance, a subsequent analysis testing whether CSF p-tau levels were directly associated with domain-specific MTL activity did not find such a relationship. This highlights the possibility that other factors than tau pathology might contribute to this imbalance in domain-specific activity.

### Increased hippocampal activity is associated with object mnemonic discrimination and CSF tau levels

In addition to extrahippocampal subregions, there are several studies that reported increased hippocampal activation accompanying difficulties in object mnemonic discrimination tasks in ApoE4 carriers (Sinha et al., 2018), healthy older individuals (Yassa et al., 2011a; Reagh et al., 2018), and MCI patients (Yassa et al., 2010; Bakker et al., 2012, 2015; Tran et al., 2016). Bakker et al. (2012, 2015) showed that a low-dose levetiracetam intervention decreased hippocampal activation levels and, as a result, improved mnemonic discrimination performance. In all these studies, increased activation in an object mnemonic discrimination task, which was mainly restricted to hippocampal DG/CA3, had seemingly detrimental effects on memory, and rodent work suggests that this might be caused by degraded inhibitory control with aging (Wilson et al., 2006). Using our task and in line with these earlier studies, we found that increased hippocampal activity in cognitively unimpaired older individuals was associated with decreased correct rejection rates in the object but not the scene condition. Interestingly, the only other study that investigated increased hippocampal activity during a combined object and spatial mnemonic discrimination paradigm in older adults found very similar results (Reagh et al., 2018). While increased activity in DG/CA3 was significantly associated with object mnemonic discrimination in healthy older adults, there was no significant relationship with object-location discrimination. This suggests that increased hippocampal activity was restricted to alEC projections.

Importantly, we investigated the relation between CSF tau and hippocampal activity and found a significant relationship where higher tau levels were associated with increased activity. Again, fluid biomarkers lack spatial specificity, and it is difficult to know whether MTL tau is underlying these increased activity levels. However, a recent study using tau PET showed that tau accumulation in inferior temporal brain regions was associated with increased hippocampal activity (Huijbers et al., 2019). Thus, tau accumulation in the inferior temporal lobe and adjacent regions may lead to dysfunction of cells in EC and PRC. Animal studies show that misfolded and hyperphosphorylated tau can impair neuronal function (Polydoro et al., 2014; Zhou et al., 2017), and this is consistent with our observation that alEC dysfunction is associated with impaired mnemonic discrimination. Early local accumulations of pathological tau in axons lead to presynaptic dysfunction, neuronal hypoactivity, and strong behavioral deficits in mice, and this effect can be rectified by restoring neuronal energy balance (Dennissen et al., 2016). Our finding that hippocampal activity correlates with tau, and therefore activity in the postsynaptic network targeted by alEC projections, indicates that the emergence of tau-related hippocampal hyperactivity in old age is input-specific and confined to inputs from alEC. The reason for this may be that we included only cognitively unimpaired individuals in this study who can be expected to have regionally very limited spread of tau pathology. It remains to be determined whether increased hippocampal activity was related to aberrant network function (Palop and Mucke, 2016) or an unsuccessful attempt to compensate for degraded inputs from alEC.

### Limitations

Our study comes with several limitations. Our object-scene fMRI paradigm was only run in a subset of the DELCODE population. As a consequence, it is important that our results will be replicated in bigger samples. Second, our current results are based on correlation analyses using cross-sectional data, and we will need to wait for the longitudinal observations from the DELCODE study to address causality. Third, our CSF-based measures do not provide spatial specificity and only allow to investigate the effect of global pathology burden on regional dysfunction. However, fMRI did help us to subscribe the effects of tau pathology to subregions in the MTL. Fourth, we were not able to stratify our sample regarding Aβ status, which leaves it open whether AD-related or non–AD-related tau pathology (PART/SNAP) causes the observed effects which we aim to achieve with a bigger dataset in the future. Finally, our resolution makes it difficult to be sure whether signal from adjacent regions contributes to the signal in our ROIs which has to be addressed in future high-resolution studies. However, we have shown that our alEC results are likely independent from signal in A35.

Together, we have shown that object mnemonic discrimination performance is associated with CSF levels of tau, but not Aβ, and that increased hippocampal activity might in part underlie this relationship. This suggests that dysfunction of the alEC-hippocampus object mnemonic discrimination network might be a marker for tau-related cognitive decline and supports the view that object mnemonic discrimination tasks could be interesting candidates for markers for cognitive decline. Future studies will be needed to investigate the profile of longitudinal cognitive decline where scene mnemonic discrimination might be predominantly impaired in later stages of AD.
